# Preventive Effects of Baclofen but Not Diazepam on Hippocampal Memory and Glucocorticoid Alterations After Prolonged Alcohol Withdrawal in Mice

**DOI:** 10.3389/fpsyt.2022.799225

**Published:** 2022-05-24

**Authors:** Henkous Nadia, Martins Fabienne, Christophe Pierard, Mons Nicole, Beracochea Daniel

**Affiliations:** ^1^Institut de Neurosciences Cognitives et Intégratives d'Aquitaine (INCIA), Université de Bordeaux, CNRS UMR 5287, Pessac, France; ^2^Institut de Recherche Biomédicale des Armées (IRBA), Place Général Valérie André, Brétigny-sur-Orge, France

**Keywords:** hippocampus, prefrontal cortex, ethanol, glucocorticoids, GABA, memory, alcohol-withdrawal

## Abstract

Our study aims at comparing in C57/Bl male mice, the impact of repeated injections of baclofen (an agonist of GABAB receptor) or diazepam (a benzodiazepine acting through a positive allosteric modulation of GABAA receptor) administered during the alcohol-withdrawal period on hippocampus-dependent memory impairments and brain regional glucocorticoid dysfunction after a short (1-week) or a long (4-week) abstinence. Hence, mice were submitted to a 6-month alcohol consumption (12%v/v) and were progressively withdrawn to water. Then, after a 1- or 4-weeks abstinence, they were submitted to a contextual memory task followed by measurements of corticosterone concentrations in the dorsal hippocampus (dHPC), the ventral hippocampus (vHPC) and the prefrontal cortex (PFC). Results showed that 1- and 4-week withdrawn mice exhibited a severe memory deficit and a significant abnormal rise of the test-induced increase of corticosterone (TICC) in the dHPC, as compared to water-controls or to mice still under alcohol consumption. Repeated daily systemic administrations of decreasing doses of diazepam (ranged from 0.5 to 0.12 mg/kg) or baclofen (ranged from 1.5 to 0.37 mg/kg) during the last 15 days of the withdrawal period, normalized both memory and TICC scores in the dHPC in 1-week withdrawn animals; in contrast, only baclofen-withdrawn mice showed both normal memory performance and TICC scores in the dHPC after a 4-week withdrawal period. In conclusion, the memory improvement observed in 4-week withdrawn mice administered with baclofen stem from the protracted normalization of glucocorticoid activity in the dHPC, a phenomenon encountered only transitorily in diazepam-treated withdrawn mice.

## Introduction

Evidence in humans and rodents have shown that alcohol-withdrawal (AW) markedly affects memory linked to hippocampal or prefrontal cortex (PFC) functional disorders and the hypothalamic-pituitary-adrenal axis (HPA) activity (1–7). Even if certain alterations induced by alcohol withdrawal on HPA axis dysfunction and fear reactivity may diminish or even disappear with time (8), studies in rodents also evidenced persistent brain regional glucocorticoids (GCs) disturbances after a chronic alcohol consumption in the PFC and the dorsal hippocampus (dHPC), up to 2 months after abstinence (9, 10). Congruently, we recently evidenced persistent working memory deficits associated with exaggerated corticosterone rises in the PFC up to 6-weeks after alcohol withdrawal in mice (11–14).

A way to reduce the HPA axis hyper-activity in abstinent subjects is to act on the GABAergic neurotransmission. Indeed, GABAergic neurons and GCs receptors (GR) have been found to be co-localized in the paraventricular nucleus of the hypothalamus, which demonstrates a critical importance to control the HPA axis activity through the GABAergic mediation (15–18). Baclofen and diazepam have an agonist action on GABAB and GABAA receptors, respectively, and are the main pharmacological treatments delivered to alcoholics during and after abstinence (19–22). These drugs have been found to reduce the HPA axis activity in withdrawn alcoholics (23–27) and to decrease addiction to alcohol both in humans and animals (19, 28–34). However, there are conflicting results on the relative efficacy of both drugs to counteract AW syndrome. In humans, both drugs induced comparable attenuation of the physical symptoms induced by AW, such as withdrawal seizures, anxiety, sweating and tremors over a 10 day withdrawal period (29) whereas a study showed a greater efficacy of the benzodiazepine chlordiazepoxide as compared to baclofen in reducing the physical symptoms of AW (35). In contrast, recent studies did not report different qualitative effects of baclofen and benzodiazepines in severe AW syndrome (36, 37). Overall, a recent review suggests that there is not enough evidence to support the use of baclofen as a first line treatment for AW syndrome (38). Most of these conflicting data have been however drawn after short periods of alcohol abstinence whereas their effects on longer periods of abstinence is lacking. So far, an unresolved issue remains to determine the relative efficacy of baclofen as regard to diazepam on protracted cognitive and GCs dysfunctions after a long period of alcohol abstinence.

As regards this issue, we implemented a mice model of long-lasting AW-induced GCs and cognitive dysfunction (12, 14). We showed that repeated diazepam administration during the withdrawal phase improved working memory and normalized GCs activity in the PFC after a short (1-Week) but not a long (6-Week) abstinence (11); in contrast, we evidenced that a sub-chronic administration of baclofen but not diazepam administered during the withdrawal phase normalized the abnormal HPA axis response to stress and reduced concomitantly the stress-induced alcohol-place preference after a long AW period, demonstrating a persistent preventing effects of baclofen but not diazepam on alcohol-seeking behavior (39).

The present study is a continuation of our previous work and aims to compare the relative efficacy of diazepam and baclofen in counteracting the persistent hippocampal-dependent memory and brain regional GCs disorders observed after AW in mice. To probe this issue, we first investigated the effects of a 1-week, (1-W) or 4-week (4-W) AW periods on memory in a serial contextual memory task known to involve the dHPC activity (40, 41). Additionally, corticosterone concentrations were quantified in the PFC, the dHPC and the ventral HPC (vHPC) of alcohol-withdrawn mice after behavioral testing. According to the data, we further compared the corrective effects of repeated systemic injections of either diazepam or baclofen administered during the withdrawal period on the protracted HPC-dependent memory dysfunction and GCs alterations in 1-W and 4-W-withdrawn animals.

## Materials and Methods

### Animals

This study has been conducted on male C57BL/6 mice (Janvier, France). Mice were 3 months old upon arrival. They were housed by groups of 10 in collective cages (425 × 276 × 153 mm; 820 cm^2^), in a temperature-controlled colony room (22 ± 1°C), under a 12:12 light-dark cycle (lights on at 7:00 a.m.). All test procedures were conducted during the light phase of the cycle between 8.00 and 12.00 a.m. During the food deprivation phase, mice (28 to 32 g) were housed individually and were maintained at 85–90% of their *ad libitum* body weight throughout the behavioral study. In both experiments 1 and 2, animals were daily handled 6 min/day during the week preceeding the beginning of the behavioral phase, to reduce the emotional reactivity to he experimenter.

### Alcohol Intake and Withdrawal Procedures

**Figure 1:**
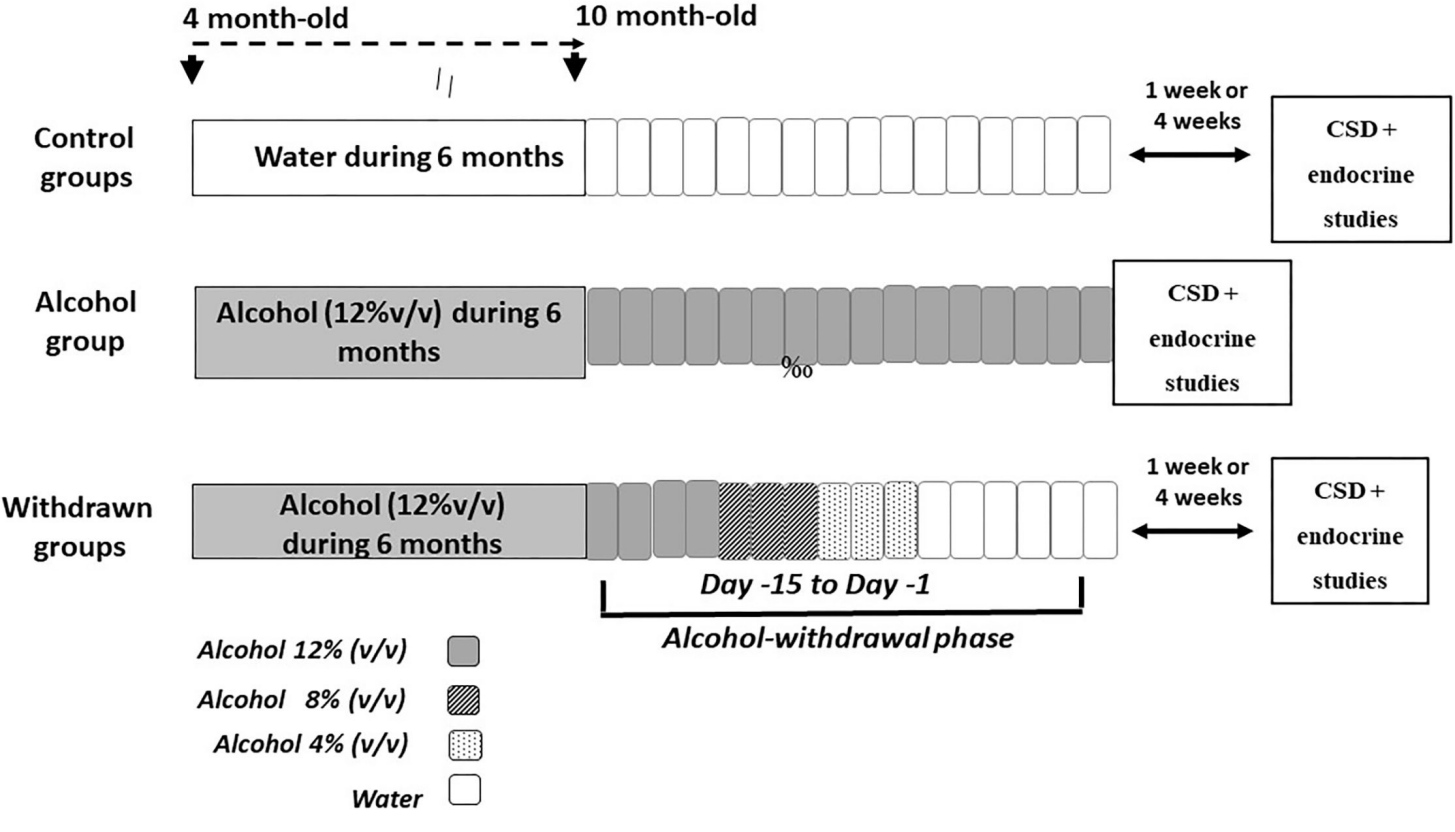
Schematic overview of the alcohol exposure and withdrawal procedures. The withdrawn groups were submitted to a 6-month exposure to alcohol (120% v/v) followed by a withdrawal phase that lasted 15 days (from Day−15 to Day−1), during which alcohol was progressively withdrawn from the solution by steps of 4% (Day−15 to Day−12: alcohol 12%; Day−11 to Day−9: alcohol 8% v/v; Day−8 to Day−6: alcohol 4%; then water for the remaining days). Either 1- or 4-weeks after the end of the withdrawal phase, mice were submitted to memory testing in the CSD task followed by the endocrine study. The alcohol group was exposed to a 6-months alcohol exposure and was still under the alcohol solution at the time of memory testing. Control groups were exposed to the same general schedule except that they received permanently water as the sole source of fluid.

The experimental device is depicted in Figure 1. At the age of 4 months, mice were given as their sole liquid source, water containing increasing concentrations of ethanol as follows: 4% (v/v) the first week, 8% (v/v) the second week and 12% (v/v) for the 6 consecutive months. After alcohol exposure, alcohol was progressively replaced by water by steps of 4% every 3 days, then water to the end of experiments. Behavioral testing began either after 1-week (1-W-withdrawn) or 4-weeks (4-W-withdrawn) of water supply (see Figure 1). The alcohol group was submitted to the same alcohol exposure as withdrawn animals, except that they were still under alcohol intake at the time of experiments. Control animals received permanently water. All procedures were performed between 8:00 and 12:00 a.m.

### Apparatus

Memory testing occurred in a four hole-board apparatus (45 × 45 × 30 cm) enclosed with gray Plexiglas walls. On the floor, 4 holes opening on a food cup (3 cm diameter × 2.5 cm in depth) were located 6 cm away from the sidewalls. Photocells located inside each hole quantified automatically the number of head-dips.

### Contextual Serial Discrimination Task

**Figure 2:**
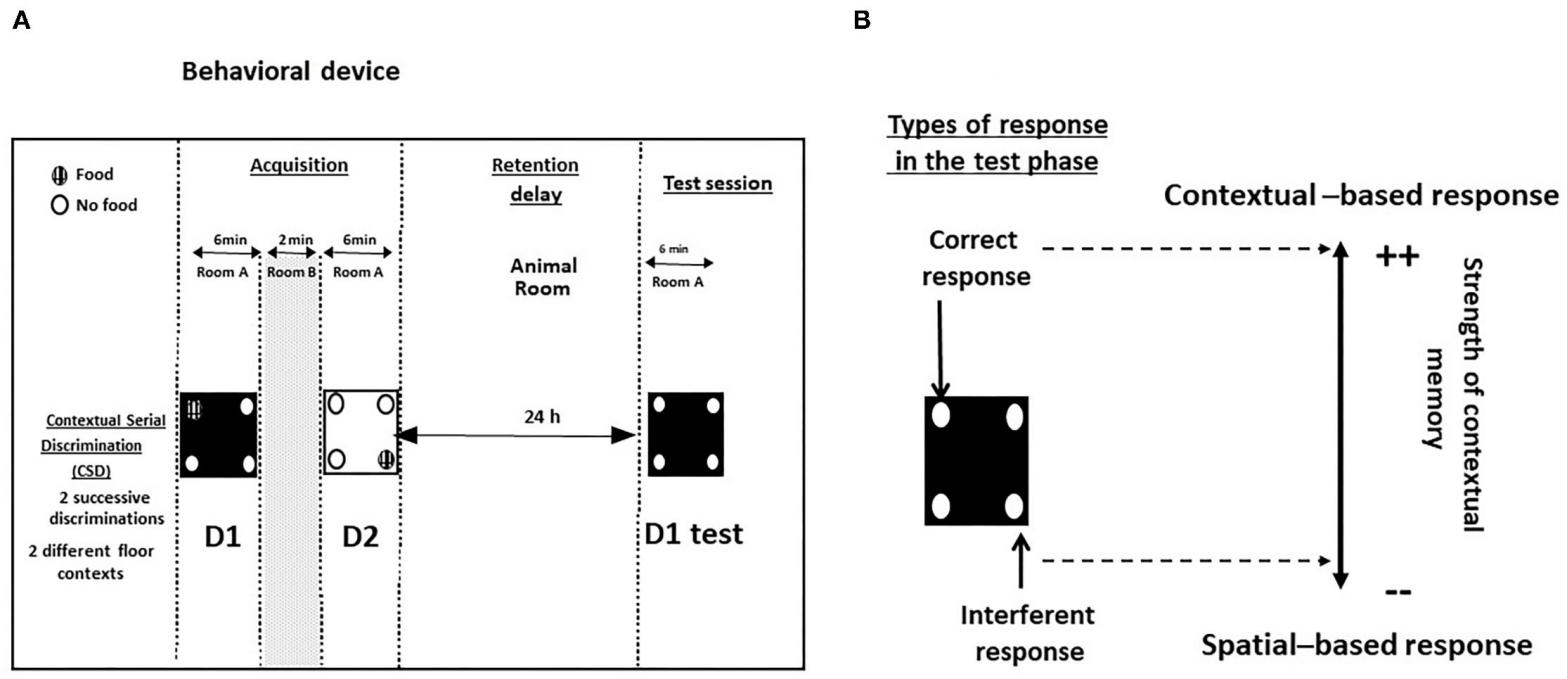
**(A)** Behavioral device for the study of contextual memory in the CSD task; **(B)** Types of responses during the test phase. Correct responses: head-dips into the hole previously baited at D1 of the acquisition phase; interferent response: head-dips into the hole baited at D2 of the acquisition phase.

The contextual and serial memory task (CSD) has been described in full in previous papers (40, 42, 43) and in Figure 2.

All mice were first food-deprived before being submitted to the behavioral test. The food deprivation procedure is aimed at increasing the motivation for the pellets used as reinforcing agents in the hole-board. To that aim, mice were given a limited quantity of food for 3 consecutive days (3 g the first day, 2 g the second day and finally 1 g the third day) before the beginning of the behavioral experiment. This food regimen induced a progressive weight loss comprised between 10 and 12% of the initial weight (before the food deprivation procedure). During this period, some pellets used in the behavioral test were placed in their cage in order to get them used to eating them.

During the acquisition phase, food-deprived mice learned two successive discriminations (D1 and D2, 6 min each) performed on two different floors differing by the color and texture (white and smooth or black and rough). The floors are alternated from one mouse to another and from one discrimination to another in order to avoid a bias related to their positioning in the series. The two serial discriminations were separated each by a 2-min time interval during which the mouse was placed in its home cage. For D1 and D2, ten 20-mg pellets (Bioserv, France) were available only in a specific hole out of the 4 holes of the device; the baited holes at D1 and D2 of the acquisition phase were changed from one mouse to another but in all cases, the baited holes at D1 and D2 were systematically opposite and symmetrical.

The retention phase occurred 24 h later, mice were replaced for 6 min on the floor used specifically at D1, with no food pellets in the apparatus. We previously showed that the memory of D1 was dependent on the dHPC activity but not on vHPC or PFC ones (40, 41, 43, 44). Two measures were taken: (1) the number of head-dips in the “correct” hole (parameter 1: head-dips into the hole previously baited on the same floor-context); (2) the number of head-dips in the “interfering” hole (parameter 2: head-dips into the hole previously baited on the other floor-context at D2). Parameters 1 and 2 allowed calculus of the “discrimination index” (% correct responses–% interfering responses). A positive difference means that mice explore more often the contextually correct hole as compared to the interfering spatial one, so that the higher the discrimination index, the more accurate is contextual memory (45).

### Pharmacological Procedure

**Figure 3:**
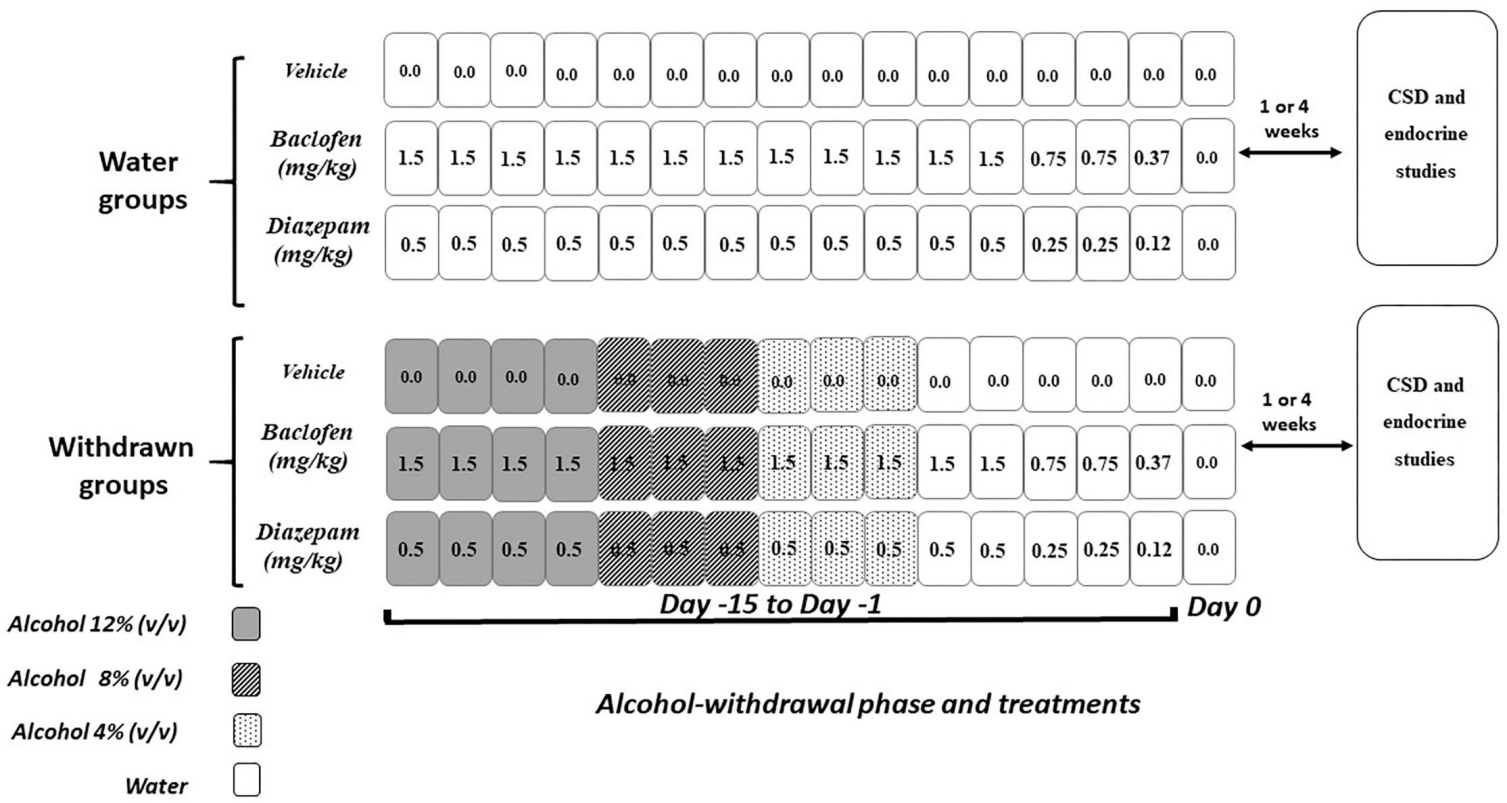
Pharmacological procedure. Diazepam and baclofen were administered by intraperitoneal injection (1/day) both in control (Water groups, upper part) and in withdrawn mice (withdrawn groups, lower part) during 15 consecutive days (Day−15 to Day−1). The pharmacological treatments started when withdrawn mice were still under the alcohol regimen (12% v/v, 4 consecutive days, dark rectangles), then 8% v/v (next 3 consecutive days gray rectangles), and finally 4% v/v (3 remaining consecutive days, light gray rectangles) followed by water (white rectangles). All mice received on the first 12 days of treatment either vehicles or diazepam at 0.5 mg/kg or baclofen at 1.5 mg/kg, followed at Days 3 and 2 by doses being half of the starting dose-−0.25 and 0.75 mg/kg, respectively, for diazepam and baclofen—and finally (Day−1) by 0.12 or 0.37 mg/kg diazepam and baclofen doses, respectively. Control groups receiving water were submitted to the same pharmacological procedures as those used in withdrawn groups. Behavioral testing and endocrinological studies occurred either 1- or 4-weeks after the last injection.

Two weeks before the pharmacological treatments, mice were housed in individual cages (331 × 159 × 132 mm; 335 cm^2^) with continuous access to alcohol. Diazepam (Valium^®^, Roche) and baclofen (Baclofen^®^, Mylan) were diluted in a vehicle solution (0.9% NaCl) and injected intraperitoneally (10 mL/kg, i.p., 1 injection/day). Drug doses were based on pilot experiments and previous studies (46, 47). In all experiments, drugs were administered over the 15 final days of the withdrawal phase while mice were still under a 12% ethanol (v/v) regimen at the beginning of the pharmacological treatments (Figure 3) according to previous studies (11, 39). Doses were progressively decreased from Day-15 to Day-1, to avoid potential negative effects of an abrupt cessation of drug administrations. Behavioral testing began either 1- or 4-weeks after the last injection. Our previous biological analyses showed that, at the time of memory testing, both diazepam and baclofen compounds were no longer detectable in the blood of treated animals and their controls (39).

### Corticosterone Assays

As shown in earlier studies, the maximum peak of corticosterone in the hippocampus or the PFC was observed 1 h after the occurrence of behavioral testing or the onset of a stressor (11, 40, 41, 48–50). Thus, in the present study, the brains were collected 1 h after behavioral testing. All mice were replaced in their individual cage in the colony room during the 1-h delay separating the end of behavioral testing and sacrifice. After this delay was elapsed, mice were briefly anesthetized (Isoflurane^®^) during a brief 30-s inhalation exposure followed by a rapid decapitation. The choice of isoflurane relies on the fact that studies have shown that this anesthetic has little or no effect on plasma corticosterone levels in male rats (51–53); in addition, the very short time between anesthesia and decapitation reduces the risk of interaction with brain corticosterone levels. After decapitation, the brains were quickly extracted according to the stereotaxic atlas of Lehmann for mouse brain (54); prefrontal cortex (from bregma +2.80 mm to +1.80 mm); dorsal hippocampi (from bregma −1.20 to −2.20 mm) and ventral hippocampi (from bregma −2.80 mm to −3.80 mm) and were rapidly dissected on ice using a brain matrix (ASI instruments, USA) to perform serial coronal slices (55); the hippocampus and cortex were snap-frozen on dry ice, then stored at −80°C.

#### Corticosterone Analyses

Tissues were homogenized with a small Dounce potter in buffer containing 300 μL RIPA Lysis Buffer, 6 μL PMSF and a protease inhibitor cocktail 1:1000 (Euromedex, France). The homogenized tissues were sonicated by ultra sounds on ice-cold (9 pulses it 5 s, amplitude 40) and centrifuged at 1,300 g for 30 min at 4°C. Supernatants were kept and stored at −80°C until quantification. Samples were prepared (dilution 1/3, 15 μL; 50 μL total) for colorimetric evaluation in a spectrophotometer (Victor, France). Corticosterone was measured using a commercial ELISA assay (Corticosterone Immunoassay, Euromedex, France).

#### Blood Alcohol Concentration Measurement

Samples (20 ul) of blood were proceeded for blood alcohol concentration using an EnzyChrom™ Ethanol Assay Kit (ECET-100, BioAssay Systems, Euromedex) according to the manufacturer's instructions.

### Statistical Analyses

Data were expressed as Means ± SEM. Analyses were performed using the Statview 5.0 software (Statistical Analysis System Institute Inc., NC, USA). The data were analyzed using one or two-ways ANOVA to determine main factor effects and their interaction, and followed by appropriate post *hoc* tests (Bonferroni/Dunnett). Correlation analyses were performed by the Spearman's correlation coefficient R. For all tests, p < 0.05 was considered statistically significant.

## Results

### Alcohol Intake and Concentration in Blood

At the end of the feed deprivation period, the weights of the animals ranged from 27.3 ± 0.8 g (minimum) to 31.7 ± 0.6 g (maximum); all mice showed a weight loss of 10% to 12% of their initial weight. Among the withdrawn groups submitted to the CSD tasks, the mean daily alcohol consumption (mL) over the 6-month alcohol exposure was 3.85 ± 0.42 mL/mouse and no significant between-groups difference was observed [*F*_(7,57)_ = 1.02; *p* = 0.14]. Thus, exposure to alcohol was considered as equivalent among the withdrawn groups. In comparison, the mean daily water consumption was measured in two groups of water controls; the mean daily water consumption was 3.1 ± 0.6 mL which did not significantly differ from alcohol-treated groups (*p* < 0.09). The blood concentration of ethanol was quantified with a commercial Elisa kit (Euromedex, France) and was 0.49 ± 0.24 g/L (10.7 ± 5.2 mM or 0.62 ± 0.30%) in mice still under the alcohol regimen in experiment 1 and below the limit of quantification in all withdrawn groups (0 ± 0 g/L) of the study at the time of memory testing.

### 1st Experiment: AW Induces Long-Lasting Contextual Memory Deficits Associated With Excessive Test-Induced Increase of Corticosterone in the Dorsal Hippocampus

In Experiment 1, independent groups of 1-W and 4-W-withdrawn mice were compared to mice still exposed to alcohol (Alcohol) and to Water-controls. Each group consists of 9 mice.

### Memory Task

#### Acquisition Phase (Data Not Shown)

ANOVA's analyses did not evidence a significant between-groups difference on the total amount of head-dips both on the first [*F*_(3,32)_ = 1.02; *p* = 0.11] and second discriminations [*F*_(3,32)_ = 0.98; *p* = 0.17]. No significant between-group difference was observed on the % number of head-dips in the baited hole of the first and second discrimination (*p* > 0.10 in both analyses).

#### Test Phase

No significant between-group difference was observed on the total number of head-dips [*F*_(3,32)_ = 0.71; *p* = 0.36; Alcohol: 47.8 ± 4.5; Water-controls: 49.3 ± 6.2; 1-W-withdrawn: 51.8 ± 3.1; 4-W-withdrawn: 47.6 ± 5.2].

**Figure 4:**
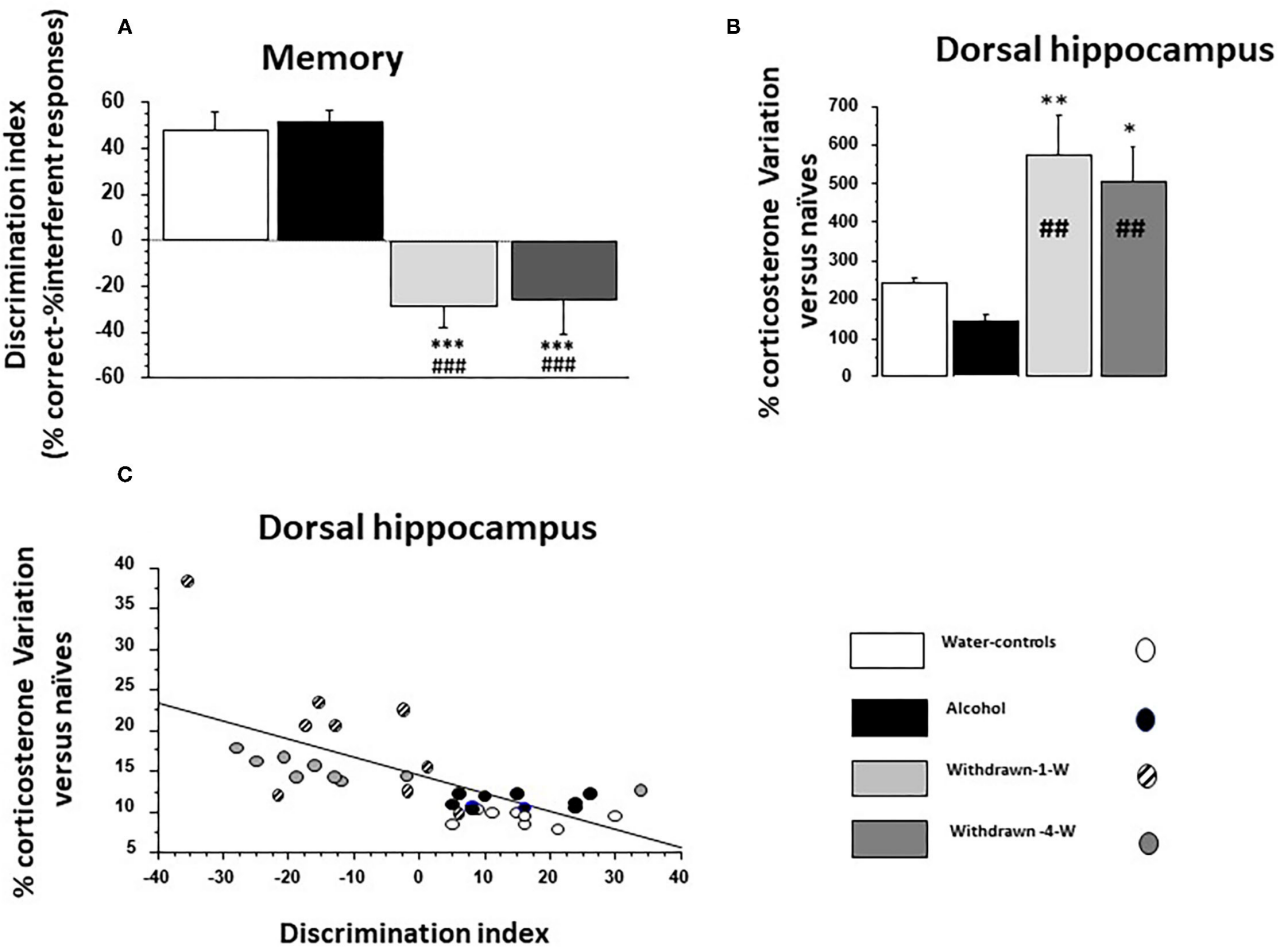
**(A)** Memory: the discrimination index is significantly lowered in both 1- and 4-W withdrawn groups as compared to both the water-control group (***p < 0.001) and the Alcohol group (###*p* < 0.001); **(B)** % test-induced increases of corticosterone concentration (TICC) in the dorsal hippocampus (dHPC) from naïve condition; as can be observed, 1- and 4-W withdrawn mice exhibited a lower TICC score as compared to water-controls (** and *: *p* < 0.01 and *p* < 0.05, respectively) and to Alcohol mice (##*p* < 0.01 in both comparisons); **(C)** Regression analysis between individual TICC score in the dHPC and discrimination index in water-controls (white circle), alcohol (black circles), 1-W (hashed gray circles), and 4-W (hashed dark gray circles) groups. A significant negative correlation was observed, the higher being the TICC score, the lower being the index discrimination.

The discrimination index was significantly different among the groups [*F*_(3,32)_ = 23.07; *p* < 0.0001; Figure 4A). Water-control and Alcohol groups exhibited a positive index [+48.09 ± 6.5% and +51.0 ± 5.3%, respectively; *F*_(1,16)_ = 0.11; *p* = 0.73] whereas 1- and 4-W-withdrawn groups showed a negative one (−28.8 ± 8.9% and −26.6 ± 13.8%, respectively; *F*_(1,16)_ = 0.019; *p* = 0.89). Both 1- and 4-W withdrawn mice differed significantly from Alcohol and Water-control groups (all *F* > 15; all ps < 0.0001).

### Brain Regional Corticosterone

#### Naïve Condition

The basal corticosterone concentration was measured in naïve mice from the four cohorts left undisturbed in their home cage during behavioral testing. Corticosterone concentrations are expressed in ng/mL in Table 1. No significant between-group differences were observed in the dHPC [*F*_(3,8)_ = 0.62; *p* = 0.61], the vHPC [*F*_(3,8)_ = 0.94; *p* = 0.46] and the PFC [*F*_(3,8)_ = 2.15; *p* = 0.17].

**Table 1:** Basal corticosterone concentrations (ng/mL) in naïve condition in the dorsal hippocampus, the ventral hippocampus and the prefrontal cortex.

	**Dorsal hippocampus**	**Ventral hippocampus**	**Prefrontal cortex**
Water-controls	3.88 ± 0.31	4.80 ± 0.50	3.70 ± 0.20
Alcohol	4.91 ± 0.39	3.84 ± 0.61	3.77 ± 0.24
1-W-withdrawn	4.38 ± 0.26	3.96 ± 0.62	3.89 ± 0.34
4-W-withdrawn	3.76 ± 1.0	4.12 ± 0.46	2.87 ± 0.45

*No significant between-group difference was observed*.

#### Test Condition

Since no significant difference was observed in basal corticosterone concentrations in the naïve condition, the test-induced increase of corticosterone concentrations (TICC) was expressed for each group as a percent variation of the naïve condition [100 × (Test - Naive)/Naive].

##### Dorsal Hippocampus

A significant between-group difference was observed [*F*_(3,10)_ = 7.72; *p* = 0.0006] (Figure 4B). The Alcohol group exhibited a lower TICC score as compared to Water-controls (+144.9 ± 16.26% vs. +242.6 ± 11.69% respectively; *p* = 0.0002). In contrast, TICC scores were significantly increased in 1W-withdrawn (+574.68 ± 103.9 %) and 4W-withdrawn groups (+505.07 ± 90.9%) as compared to Water-controls (*p* = 0.009 and *p* = 0.016, respectively) and to the Alcohol group (*p* = 0.0016 and *p* = 0.0022, respectively).

##### Ventral Hippocampus

ANOVA analyses evidenced no significant between-group difference [*F*_(3,10)_ = 2.31; *p* = 0.09]. Both the Water-control and Alcohol groups exhibited comparable TICC scores (+239.07 ± 24.6% and +260.4 ± 38.48%, respectively) which were however higher in 1W-withdrawn (+445.5 ± 86.8%) and 4W-withdrawn groups (+396.14 ± 80.5%).

##### Prefrontal Cortex

No significant between-group difference was observed [*F*_(3,10)_ = 2.59; *p* = 0.07]. Alcohol mice (+295.18 ± 20.91%) exhibited a higher TICC score as compared to Water-controls (+192.5 ± 23.35%) which was increased in 1W- and 4W-withdrawn groups (+421.35 ± 68.21% and +373.78 ± 89.98 %, respectively).

### Correlation Analyses

#### Dorsal Hippocampus

The discrimination index correlated negatively with TICC scores (Figure 4C; R = −0.67; *p* < 0.0001). More specifically, Water-control and Alcohol mice exhibited a high discrimination index associated with low TICC scores; in contrast, 1W and 4W-withdrawn groups exhibited a low discrimination index associated with high TICC scores.

#### Ventral Hippocampus

No significant correlation was observed between the discrimination index and TICC scores (R = 0.14; *p* > 0.10).

#### Prefrontal Cortex

No significant correlation was observed between the discrimination index and TICC scores (R = 0.21; *p* > 0.10).

### Experiment 2. Baclofen but Not Diazepam Reversed the Long-Term Memory Impairments and GCs Alterations in Withdrawn Mice

Experiment 2 was run using 12 groups of mice: 1- and 4-W withdrawn mice receiving either vehicle, diazepam or baclofen (*N* = 8 for all groups except 1-W diazepam: *N* = 7) and their respective water controls (*N* = 8 for all groups except 1-W water control injected with vehicle or baclofen: both *N* = 7).

### Memory

#### Acquisition Phase (Data Not Shown)

No significant between-group differences were observed on the total number of head-dips and the % of head-dips in the baited holes of acquisitions 1 and 2 (*p* > 0.10 in all analyses).

#### Test Phase

**Figure 5:**
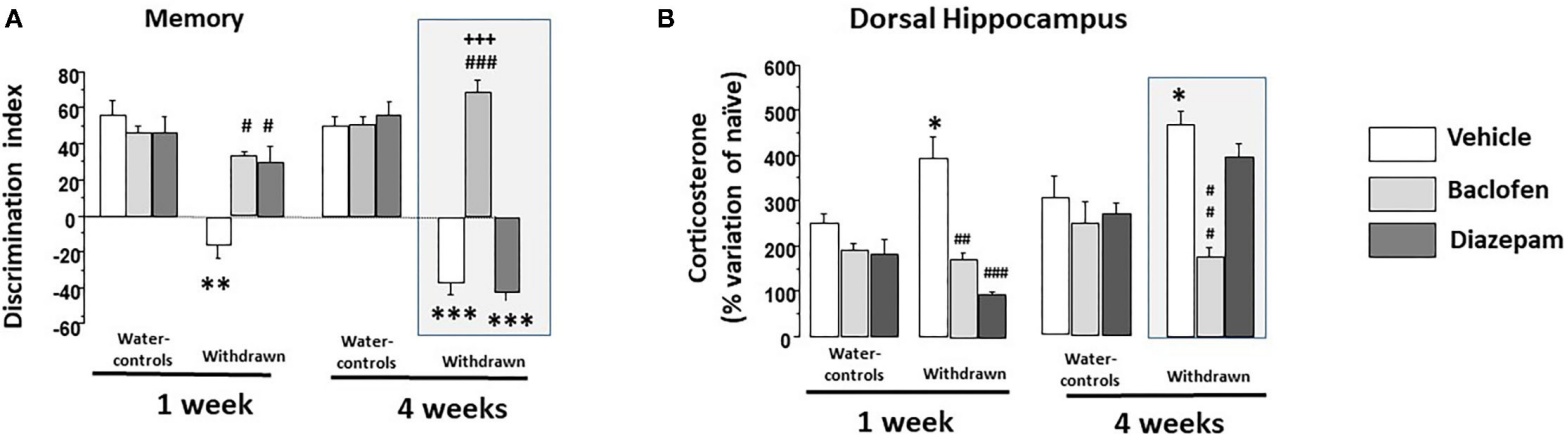
**(A)** Memory: in 1-W groups (left part), water-controls being injected either with vehicle (white square), diazepam (light gray square) or baclofen (dark gray square) exhibited high and similar discrimination indexes; in contrast, 1-W vehicle-withdrawn mice exhibited a significant negative discrimination index as compared to the vehicle-water group (**p < 0.01). 1-W withdrawn groups treated with diazepam or baclofen did not significantly differed from vehicle-water controls (*p* > 0.10 in each comparisons), which were significantly higher than that of the vehicle-withdrawn group (##*p* < 0.01 in both comparisons); in 4-W groups (right part), water-controls treated with vehicle or diazepam or baclofen exhibited high and similar discrimination indexes; in contrast, 4-W vehicle or diazepam-withdrawn mice exhibited significant negative discrimination indexes as compared to the vehicle-water group (***p < 0.001 in both comparisons). In contrast, baclofen-withdrawn mice exhibited performance similar to those of vehicle-water controls which were significantly higher than that of the vehicle-withdrawn and diazepam-withdrawn groups (###*p* < 0.001 in both comparisons); **(B)** Test-induced increase of corticosterone concentration (TICC) in the dorsal hippocampus (dHPC) from naïve condition; as can be observed, 1- and 4-W vehicle-withdrawn mice exhibited a higher TICC score as compared to respective vehicle-Water-controls (*p < 0.05 in each comparison). Both diazepam and baclofen 1-W-withdrawn mice exhibited a significant attenuation of the TICC score as compared to vehicle -withdrawn 1-W mice (### and ##: *p* < 0.001 and *p* < 0.01, respectively); in contrast however, only baclofen-withdrawn mice still exhibited a significant attenuation of the TICC score in 4-W-withdrawn animals as compared to both vehicle-treated and diazepam-treated withdrawn groups (###*p* < 0.001 in both comparisons). ^#^*p* < 0.05.

Data are depicted in Figure 5A. A two-way ANOVA showed significant effects of groups (withdrawn 1- and 4-W, vehicles 1- and 4-W; *F*_(3,81)_ = 56.02; *p* < 0.0001), drugs (vehicle, baclofen and diazepam; *F*_(2,81)_ = 43.53; *p* < 0.001) and the interaction between groups and drugs was also significant [*F*_(6,81)'_ = 28.1; *p* < 0.0001].

##### 1-W-Withdrawn and Water-Control Groups

A two-way ANOVA showed significant effects of groups (withdrawn-1-W and vehicles-1-W; *F*_(1,40)_=32.36; *p* < 0.0001), drugs (vehicle, baclofen and diazepam; *F*_(2,40)_ = 9.03; *p* < 0.0006) and the interaction between groups and drugs was also significant [*F*_(2,40)_ = 9.07; *p* < 0.0006]. Vehicle-withdrawn mice exhibited a lower discrimination index (-16.20 ± 7.3) as compared to vehicle-Water-controls (+46.69 ± 5.9; *p* < 0.0001). Both 1-W-withdrawn diazepam (+29.37 ± 8.86) and 1-W-withdrawn baclofen (+35.04 ± 2.30) groups exhibited higher discrimination indexes as compared to vehicle-withdrawn mice (*p* = 0.0005 and *p* < 0.0001, respectively). In contrast, they did not statistically differ from vehicle-Water-controls (*p* = 0.076 and *p* = 0.13, respectively). In water-controls, diazepam (+46.51 ± 8.93) or baclofen (+46.71 ± 3.38) did not modify the discrimination index as compared to the vehicle-Water-control group (*p* = 0.97 and *p* = 0.78, respectively).

##### 4-W-Withdrawn and Water-Control Groups

A two-way ANOVA showed significant effects of groups [withdrawn-4-W and vehicles-4-W; *F*_(1,41)_ = 145.5; *p* < 0.0001], drugs (vehicle, baclofen and diazepam; *F*_(2,41)_ = 57.19; *p* < 0.0001] and the interaction between groups and drugs was also significant [*F*_(2,41)_ = 64.87; *p* < 0.0001]. Vehicle-withdrawn mice exhibited a lower discrimination index as compared to vehicle-Water-controls (−37.42 ± 6.76 and +52.35 ± 5.35, respectively; *p* < 0.0001). Baclofen-withdrawn mice exhibited a higher discrimination index (+69.08 ± 5.79) as compared to vehicle-withdrawn mice (*p* < 0.0001) which was not observed in diazepam-withdrawn mice (−42.22 ± 4.46; *p* = 0.56 vs. vehicle-withdrawn mice). Baclofen-withdrawn mice did not statistically differ from vehicle-Water-controls (*p* = 0.052) but significantly differed from diazepam-withdrawn mice (*p* < 0.0001); in contrast, diazepam-withdrawn mice still exhibited a significant lower discrimination index as compared to vehicle-Water-controls (*p* < 0.001). Diazepam and baclofen Water-control groups (+55.73 ± 7.43 and +50.66 ± 4.30, respectively) did not statistically differ from vehicle ones (*p* = 0.71 and *p* = 0.80, respectively).

### Brain Regional Corticosterone Concentrations

Insofar as the correlation between the discrimination index and TICC score was statistically significant only with dHPC corticosterone scores, the endocrinal studies in experiment 2 were performed in the dHPC only.

#### Naïve Condition

Data are expressed in Table 2. No significant between-group difference was evidenced observed [*F*_(11,36)_ = 1.24; *p* > 0.10].

**Table 2:** Basal corticosterone concentrations (ng/mL) in naïve condition in the in the dorsal hippocampus.

**Groups**	***N***	**Corticosterone in ng/mL**
Vehicle Water-controls 1-week	4	4.23 ± 0.30
Baclofen Water-controls 1-week	4	4.26 ± 0.77
Diazepam Water-controls 1-week	4	4.12 ± 0.64
Vehicle 1-week-withdrawn	4	3.97 ± 0.49
Baclofen 1-week-withdrawn	4	4.21 ± 0.58
Diazepam 1-week-withdrawn	4	3.64 ± 0.69
Vehicle Water-controls 4-week	4	3.90 ± 0.40
Baclofen Water-controls 4-week	4	4.08 ± 0.61
Diazepam Water-controls 4-week	4	4.00 ± 0.85
Vehicle 4-week-withdrawn	4	4.20 ± 0.38
Baclofen 4-week-withdrawn	4	3.60 ± 0.52
Diazepam 4-week-withdrawn	4	4.35 ± 0.53

*No significant between-group difference was observed*.

#### Test Condition

Since no significant difference was observed in basal corticosterone concentration in the naïve condition, the test-induced increase of corticosterone concentrations (TICC) was expressed for each group as a percent variation of the naïve condition [100 × (Test - Naive)/Naive].

Data are depicted in Figure 5B. A two-way ANOVA showed significant effects of groups [withdrawn 1- and 4-W, vehicles 1- and 4-W; *F*_(3,81)_ = 12.34; *p* < 0.0001], drugs [vehicle, baclofen and diazepam; *F*_(2,81)_ = 27.12; *p* < 0.001] and the interaction between groups and drugs was also significant [*F*_(6,81)_ = 7.73; *p* < 0.0001].

In the 1-Week groups, a two-way ANOVA showed no significant effects of groups [withdrawn-1-W and vehicles-1-W; *F*_(1,40)_ = 0.40; *p* = 0.52], but a significant effect of drugs [vehicle, baclofen and diazepam; *F*_(2,40)_ = 23.89; *p* < 0.0001] and the interaction between groups and drugs was also significant [*F*_(2,40)_ = 9.53; *p* < 0.0004]. More specifically, vehicle 1-W-withdrawn mice (+394.6 ± 46.6%) exhibited a significant increase of TICC scores as compared to respective Water-control mice (+248.6 ± 24.58%; *p* = 0.019). Diazepam-1-W-withdrawn mice exhibited a significant reduction of TICC scores as compared to vehicle-withdrawn mice (+98.2 ± 6.1%; *p* < 0.0001); a similar reduction of TICC scores was also observed in baclofen-treated withdrawn mice (+173.8 ± 13.2%; *p* < 0.0004 vs. vehicle 1-W-withdrawn mice).

In the 4-week groups, a two-way ANOVA showed significant effects of groups [withdrawn-4-W and vehicles-4-W; *F*_(1,41)_ = 6.97; *p* < 0.011], drugs [vehicle, baclofen and diazepam; *F*_(2,41)_ = 13.38; *p* < 0.0001], and the interaction between groups and drugs was also significant [*F*_(2,41)_ = 6.87; *p* < 0.0027]. More specifically, Vehicle 4-W-withdrawn mice exhibited a significant increase of the TICC scores as compared to respective Water-controls (+468 ± 29.01% and +301.01 ± 50.4%, respectively; *p* = 0.012) which was significantly lowered in baclofen (+175.7 ± 29.01%) but not in diazepam-treated withdrawn groups (+398.37 ± 25.9%; *p* < 0.0001 and *p* = 0.09, respectively, vs. Vehicle-4-W-withdrawn mice). In addition, baclofen-withdrawn mice differed significantly from diazepam-withdrawn ones (*p* < 0.0001).

### Correlation Analyses

ANOVA analyses performed in all groups within Experiment 2 showed a significant negative correlation between the discrimination index and the TICC score (R = −055; *p* < 0.0001). More specifically, the higher is the discrimination index, the lower is the TICC score.

#### 1-W Groups

**Figure 6:**
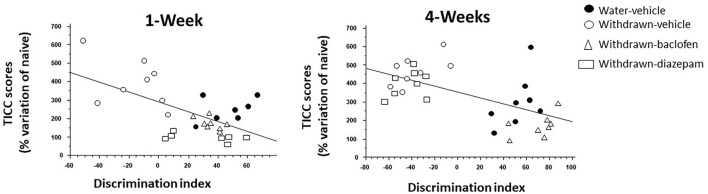
Regression analysis between individual dHPC TICC scores and discrimination indexes in vehicle-Water-controls (hashed squares), withdrawn-vehicle (hashed triangles), diazepam-withdrawn (white circle) and baclofen-withdrawn (black circles) groups. (left): in 1-W groups, a significant negative correlation was observed, the higher being TICC score, the lower being the index discrimination. As can be observed, 1-Week vehicle-withdrawn mice exhibited the lower discrimination index and the higher TICC scores among the 4 groups (right) in 4-Week withdrawn mice, a similar negative correlation was also observed, but in contrast to 1-W withdrawn mice, diazepam-treated withdrawn mice exhibited similar endocrinal and behavioral patterns as vehicle-withdrawn animals.

The discrimination index correlated negatively with TICC scores (R = −0.65; *p* < 0.0001). Interestingly, as shown in Figure 6 (left), baclofen and diazepam-treated mice exhibited a high discrimination index and a low TICC score similar to vehicle-controls, whereas vehicle-withdrawn mice exhibited an opposite pattern, that is to say a high TICC score and a low discrimination index.

#### 4-W Groups

The discrimination index correlated negatively with TICC scores (R = −0.57; *p* < 0.0001). Interestingly, as shown in Figure 6 (right), baclofen-treated mice exhibited a high discrimination index and a low TICC score similar to vehicle-controls; in contrast to what was observed in 1-W animals however, diazepam-treated mice exhibited an opposite pattern, that is to say a high TICC score and a low discrimination index, therefore similar to that of vehicle-withdrawn mice.

### Discussion

Our study aims at comparing the impact of repeated injections of baclofen or diazepam administered during the alcohol-withdrawal period on hippocampus-dependent memory impairments and brain regional GCs dysfunction after a short (1-W) or a long (4-W) abstinence from alcohol. Results showed that both withdrawn groups exhibited contextual memory deficits and significant abnormal rise of the test-induced increase of corticosterone specifically in the dHPC compared to water controls. Interestingly, while both chronic diazepam or baclofen treatment during the withdrawal period rescued contextual memory deficits and prevented the test-induced GCs alterations in 1-W withdrawn animals, only baclofen-treated animals showed memory performance and test-induced rise of corticosterone similar to those of water-controls after a 4-Week withdrawal period.

### Alcohol Intake

A prime issue to be addressed rested with assessing whether the memory and endocrine alterations observed in alcohol-withdrawn mice may be caused by differences in diets. Findings evidenced that differences in the daily amount of food consumption may not be held accountable for the deficits since we have already demonstrated that pair-fed animals receiving an isocaloric solution of dextri-maltose during the same duration (6 months period) of alcohol exposure exhibited no memory deficits (56). Moreover, since most mice strains exhibit low appetence for alcohol, they often restrain their daily liquid intake and exhibit signs of dehydration. Such was not the case in our study, since the C57BL/6 strain is an alcohol-preferring strain (57); further, mice submitted to alcohol intake drank a slightly higher daily amount of liquid solution as compared to water controls. Thus, alcohol-withdrawn mice were not dehydrated during alcohol exposure. Finally, the daily alcohol intake during alcohol exposure in the different withdrawn groups was similar: hence, we may legitimately infer that all groups were equally exposed to alcohol, thus allowing for valuable comparisons among the different cohorts.

Moreover, it has been already reported that GABAB and GABAA receptor agonists modulate alcohol drinking (58, 59) and even might have an opposite effect on alcohol drinking as they have for food intake (60).In the present study however, the interaction between alcohol drinking and GABA agonists injections occurred only during the first 10 days of the withdrawal phase, during which the alcohol concentration was progressively decreased to tap water. It is very unlikely that such a short interaction period had any impact on the long-lasting cognitive dysfunction observed in alcohol withdrawn animals.

### Alcohol-Withdrawal Produced Long-Lasting Memory Impairments and Brain Regional GCs Dysfunction in the Dorsal Hippocampus

In the present study, we did not directly measure locomotor activity in both the acquisition and test phases of the task. It is known that alcohol consumption and withdrawal may induce side effects such as an alteration of locomotor activity, even though contrasting data are reported according to the duration of the treatments and withdrawal period (see in 60). However, since alterations in locomotor activity can potentially alter the performance in the memory task, one cannot exclude that the lack of measurement of this parameter may be a limitation to the study. Nevertheless, substantial data allow us to rule out this hypothesis. First, we did not observe in the present study a significant difference in the exploration of the total number of head-dips in the baited and unbaited holes among the groups during the acquisition phase: such a finding infers that locomotor activity is similar in all groups. This observation confirmed previous experimental data showing transitory impairments in exploring a 9 hole-board only in 1-week but not in 6-weeks withdrawn mice, as compared to controls (61). In addition, we previously showed that locomotor activity was spared in withdrawn mice in several other tasks. More specifically, we elicited a normal sequential alternation behavior over 6 successive trials separated by a short intertrial interval, as well as normal choice latencies between arms in a T-maze in 6-weeks alcohol withdrawn mice, as compared to controls (11); we also evidenced that the total number of entries in open and closed arms of an elevated plus-maze was similar in alcohol-withdrawn mice as compared to controls (11) as well as the total number of crossed sections (an index of locomotor activity) in an open-field or in an open area in an odor recognition task in a non-stress condition (39). Thus, in our experimental conditions, an alteration of locomotor activity is unlikely responsible for the cognitive recognition deficits in withdrawn animals.

Our present findings agree with other studies showing that AW produced sustained alterations of memory in alcohol-withdrawn rodents as compared to water-controls or to rodents still under alcohol regimen (7, 13, 43).

From a psychological point of view, exaggerated anxiety levels resulting from alcohol-withdrawal have been considered as a potential key factor of the long-lasting maintenance of cognitive deficits over time. Indeed, human and rodent studies have reported enhanced anxiety-like behaviors in various tests during ethanol withdrawal (62). In our experimental conditions, previous studies evidenced however only a mild increased anxiety-like reactivity in an elevated plus-maze and open-field tasks, and very mild withdrawal symptoms in 1-Week withdrawn animals, which however were not observed in 6-Weeks withdrawn mice (11, 39). In these studies, we showed that circulating and basal brain regional corticosterone levels are not modified by alcohol-withdrawal, as compared to Water-controls. These endocrinal data fit well with the mild anxiety symptoms observed in withdrawn animals. Nevertheless, our findings contrast with other studies showing that withdrawn animals and humans exhibit potentially elevated corticosterone levels during the acute withdrawal phase and that prolonged withdrawal and abstinence are rather characterized by a blunted GC response over time (2). Several factors may, however, account for the discrepancies. Firstly, the withdrawal procedure implemented in our study was not abrupt since the amount of alcohol in the solution was gradually reduced down to water over the 15 days of the withdrawal phase. Such a gradual withdrawal procedure is likely to have induced a reduced emotional reactivity to the withdrawal; secondly, animals in our studies were evaluated for emotional reactivity at least 1-week after withdrawal, rather than in the immediate wake of alcohol intake cessation; such a procedure can attenuate the anxiety-like symptoms often observed after an abrupt alcohol withdrawal. Another factor can also be linked to the strain of mouse used, insofar as C57BL/6 is an alcohol-preferring strain (57) whose HPA axis responses to alcohol and stress differ from other mouse strains (63–65). Given that, an increase of anxiety is unlikely responsible for the contextual memory impairments and GCs dysfunction observed after prolonged alcohol-withdrawal in our experimental conditions.

Endocrine studies in humans (1, 2, 4, 6) and rodents (3, 5, 66) have shown that AW markedly affects the HPA activity. Studies in rodents evidenced persistent brain regional GCs disturbances after a chronic alcohol consumption in the PFC and the dHPC, up to 2 months after abstinence (9, 13). A prime result of this study is to show that the contextual memory impairments observed in alcohol-withdrawn mice are linked with an excessive and significant test-induced corticosterone rise specifically in the dHPC. Indeed, correlation analyses evidenced a significant negative interaction between corticosterone scores in the dHPC and the memory discrimination index, the higher being corticosterone levels, the lower being memory performance. In addition, these findings are in accordance with our previous studies having shown that injections of corticosterone into the dHPC in normal (non-alcoholics) mice induced a severe memory impairment in the CSD task similar to that observed here in alcohol-withdrawn animals (40, 43); moreover, these studies also showed that blocking the stress-induced increase of corticosterone in the dHPC by a systemic administration of metyrapone (an inhibitor of corticosterone synthesis) prior the onset of a stressor canceled out both the contextual memory deficits and the stress-induced rise of corticosterone in the dHPC (40). Based on these findings, it can be assumed that the abnormal test-induced rise of corticosterone in the dHPC is responsible for the memory impairments observed in 1-W and 4-W withdrawn mice in the CSD task.

Interestingly, the vHPC and the PFC exhibited non-significant enhancements of the TICC scores as compared to water-controls. We previously showed that these brain areas are not recruited in the processes sustaining memory of the first discrimination in the CSD task (41, 43, 44, 67). In contrast, close relationships have been previously observed in 6-week alcohol-withdrawn mice between working memory impairments in a T-maze and TICC scores in the PFC, but not in the dHPC (13). These data suggest that the magnitude of TICC rise within a given brain region may depend on its functional recruitment in the task.

### Baclofen but Not Diazepam Cancels Out the Protracted Memory and GCs Alterations Induced by Alcohol-Withdrawal

In the present study, diazepam and baclofen were undetectable in the blood of animals at the time of behavioral testing (39) and therefore, their beneficial impacts cannot be ascribed to an acute effect at the time of memory testing.

We reported here-above that repeated diazepam administrations counteract only transitorily the memory impairment and neuroendocrine disorders in 1-W-withdrawn but not in 4-W-withdrawn mice. We previously evidenced corrective effects of a similar repeated diazepam administration on working memory alterations after a short (1-week) but not a long (6-weeks) withdrawal period (13). The failure of repeated diazepam administration to counteract the persistent cognitive and neurobiological disorders in 4-W-withdrawn mice may stem either from persistent alterations of GABAA receptors (68–70), increased downregulation of the GABAA receptors over repeated diazepam administration (71) or other alcohol-induced functional and structural neuroadaptations that may progressively develop over time after withdrawal, such as alterations of epigenetic mechanisms (12, 72, 73). Indeed, chronic exposure to alcohol produces brain adaptive changes in several neurotransmitter systems, including GABA, glutamate, and norepinephrine pathways (74) in order to compensate for alcohol-induced destabilization and restore neurochemical equilibrium (75). In particular, in different rodent models of alcohol addiction, a reduction in number, function, and sensitivity to GABA of the GABAA receptors have been reported (70, 76–79) as well as alterations of plasticity between synaptic and extrasynaptic receptors (80, 81). These alterations can, in turn, reduce the efficacy of diazepam to counteract the protracted alterations of the HPA axis activity in withdrawn mice.

In sharp contrast, baclofen suppressed the protracted memory impairments and normalized the test-induced increase of corticosterone in the dHPC regardless the withdrawal periods, showing thereby prolonged counteracting effects of baclofen on these impairments. Interestingly, the beneficial effects of baclofen on alcohol addiction and relapse in abstinent subjects as well on HPA axis disorders and protracted cognitive impairments have been well substantiated (14, 39, 82). Thus, Geisel et al. (27) evidenced in abstinent alcoholics increased plasma GC levels, which were decreased in baclofen-treated patients, up to 14-weeks after treatment. In addition, Jacquot et al. (10) showed that the administration of mifepristone (an antagonist of the progesterone and GCs receptors) in mice given at the time of alcohol withdrawal totally suppressed the memory impairments in several recognition tasks up to 2-weeks after alcohol abstinence. According to these authors, the action of mifepristone suggests that GCs during the acute withdrawal phase are important in triggering the subsequent changes in neuronal function responsible for memory deficits during the abstinence period. It is hypothesized that the raised GCs concentrations during the acute withdrawal period are likely to contribute to the above changes in neuronal plasticity seen after withdrawal from chronic alcohol treatment and to the memory deficits. In view of the long-time interval (1- to 2-weeks) between the administration of mifepristone and its protracted effects, it is possible that the drug could have produced its effects by preventing long-lasting changes in the expression of genes that trigger adaptive changes in HPA axis regulation during alcohol withdrawal. More specifically, Huang et al. (83) showed in knock-out mice lacking for the FKBP5 gene (a gene intervening in the negative feedback on HPA axis function) an enhanced sensitivity to alcohol withdrawal.

Since several studies evidenced different HPA axis responses to alcohol consumption and withdrawal in female as compared to male rodents (84, 85), our present findings may indeed be restricted to male mice only. Thus, an extended development to our current study would involve the following stakes, namely, to determine whether similar alcohol-induced endocrine and memory disorders are to be observed in female mice and, further, to assess whether baclofen and diazepam bear similar counteracting effects on such disorders.

## Conclusion

Overall, the present study provides evidence that acting on the GABAB receptor through repeated baclofen administration during the alcohol-withdrawal phase counteracted the persistent hyper-reactivity of HPA axis to behavioral testing in the dHPC and rescued memory found to be altered up to 4-weeks after the cessation of alcohol intake; in contrast, diazepam, a compound having an agonist action on the GABAA receptor, induced only a transitory beneficial effect on the memory and endocrinal alterations.

## Data Availability Statement

The raw data supporting the conclusions of this article will be made available by the authors, without undue reservation.

## Ethics Statement

The animal study was reviewed and approved by EU Directive 2010/63/EU and by the local Ethical Committee of Bordeaux (#5012089).

## Author Contributions

HN and MF were equally involved in behavioral and neuroendocrine studies. MN, CP, and BD were involved in data and statistical analyses, the experimental design, and writing of the article. All authors have approved the final article.

## Funding

This research was supported by a grant from FRA (Fondation pour la Recherche en Alcoologie, FRA, Paris France attributed to BD and MN) and the CNRS. We thank Dr. Frances Ash for language proofreading (frances.ash@orange.fr).

## Conflict of Interest

The authors declare that the research was conducted in the absence of any commercial or financial relationships that could be construed as a potential conflict of interest.

## Publisher's Note

All claims expressed in this article are solely those of the authors and do not necessarily represent those of their affiliated organizations, or those of the publisher, the editors and the reviewers. Any product that may be evaluated in this article, or claim that may be made by its manufacturer, is not guaranteed or endorsed by the publisher.
